# Oocyte Positional Recognition for Automatic Manipulation in ICSI

**DOI:** 10.3390/mi9090429

**Published:** 2018-08-25

**Authors:** Mozafar Saadat, Amir M. Hajiyavand, Ajai-pal Singh Bedi

**Affiliations:** Department of Mechanical Engineering, School of Engineering, University of Birmingham, Birmingham B15 2TT, UK; m.saadat@bham.ac.uk (M.S.); ajaisinghbedi@gmail.com (A.-p.S.B.)

**Keywords:** micromanipulation, oocyte, polar body, detection, image processing

## Abstract

Polar body position detection is a necessary process in the automation of micromanipulation systems specifically used in intracytoplasmic sperm injection (ICSI) applications. The polar body is an intracellular structure, which accommodates the chromosomes, and the injection must not only avoid this structure but be at the furthest point away from it. This paper aims to develop a vision recognition system for the recognition of the oocyte and its polar body in order to be used to inform the automated injection mechanism to avoid the polar body. The novelty of the paper is its capability to determine the position and orientation of the oocyte and its polar body. The gradient-weighted Hough transform method was employed for the detection of the location of the oocyte and its polar body. Moreover, a new elliptical fitting method was employed for size measurement of the polar bodies and oocytes for the allowance of morphological variance of the oocytes and their polar bodies. The proposed algorithm has been designed to be adaptable with typical commercial inverted microscopes with different criteria. The successful experimental results for this algorithm produce maximum errors of 5% for detection and 10% for reporting respectively.

## 1. Introduction

Positioning and orienting the polar body in conventional intracytoplasmic sperm injection (ICSI) is controlled by an embryologist. Currently, cell manipulation in conventional ICSI has been manually operated by well-trained embryologists. The oocyte is manipulated by using a micropipette and inducing negative pressure to hold and relief the oocyte repeatedly until the polar body is positioned in the desired location. This type of manipulation is known as a trial-and-error course of action [1]. During the conventional operation, a single cell is manipulated several times until it is located in the desired position and orientation preparing it for injection. In cell manipulation, researchers have attempted to automate this process. There are various research projects focusing on automating 3D manipulations of biological cells [2,3,4,5]. Robotic cell manipulation with a high success rate and degree of accuracy has been reported in some publications [4,6,7,8]. However, there are some deficiencies introduced in each method, such as lack of correct detection of oocyte. Different methods of manipulations have been reported in research, which are lab-on-a-chip using microfluidic flow advances [9,10,11,12,13], optical tweezers [14], and micromechanical grippers [15,16,17,18].

Cell detection is an essential method towards 3D cell manipulation. In this paper’s proposed application, image processing techniques are utilised foroocyte and polar body recognition. The first attempt at a vision detection algorithm for cell manipulating purposes was designed to use morphological factors and Bayesian assessment as a classifier of texture and shape [19]. The major problem with this method is that it is computationally expensive. Another method, the Hough cell detection algorithm (HCDA) utilized high pass filtering to detect the edges of the holding pipette and determine the oocyte size value [12]. In this method, polar body detection was not developed. Again, the computational time for this method is high for a real time application which was 22 seconds for detection. Different algorithms have been proposed for detecting the polar body during the manipulation. However, an early detection algorithm recognized the polar body only when in plane [20]. This method involves the Otsu adaptive algorithm and morphological operator for fitting the circle to the cell. Otsu is a clustering based image threshold technique employed to convert the gray scale image to a binary image. A linearization method was employed here for polar body recognition [21]. The failure of this technique happens when the gray scale of the polar body and the oocyte are in the same level of surroundings in the image. Later, an algorithm was added to this method that estimated the polar body location out of plane using frame-by-frame motion analysis [20].

Image binarization is a method developed to detect the polar body. However, this method is highly dependent on the gray level of the neighborhood pixels in the image. Inaccuracy occurs when the gray levels are similar in the surrounding pixels [12]. Another method of polar body detection was based on the polar body’s texture data. Oocyte texture deficiencies and microscope light regulation limitation are two main disadvantages of this technique, which cause low detection accuracy [22]. Polar body and oocyte detections were proposed based on a machine learning method using image classification. In this method, the improved histogram of oriented gradient (HOG) algorithm is used to extract features of polar body images for the prediction of the polar body position in the image, determination of this position, and finally, detection [23,24]. Recently, a method has been proposed for oocyte and polar body detection that employs template matching followed by morphological operations and thresholding [5]. The method is computationally expensive based on the sizes of the template and may require new templates for morphological variance of oocytes.

Visual detection algorithms for cell injection automation have involved the Hough transform to detect the polar body [25], roundness and elongation calculations following segmentation to detect the oocyte and injector respectively [26], and a connected neighborhood method for oocyte detection [27].

The circular gradient weighted Hough transform was chosen for its simplicity and robustness for circular detection. Gradient weighting was utilized to improve circular detection of the oocyte when images suffered from blurring. The Hough transform can be computationally expensive when radius ranges are not known. In this instance the radius range was known based on typical oocyte sizes and image scale making this an efficient and powerful detection method. Other methods including template matching can be computationally expensive [19]. It may also require new templates for variation in oocytes and connected neighborhood methods for the oocyte detection [28], which required manual selection of the oocyte contour as a starting point. This would not be required when utilizing the circular gradient weighted Hough transform.

A new detection technique for the oocyte and polar body has been proposed by our group which is developed using images detection rather than continuous frame tracking using video and employs a gradient weighted Hough transform in the application of ICSI [29].

The aim of this paper is to discuss the previously mentioned methods and the process of its development. Image processing techniques will be discussed initially and then the proposed detection method will be discussed as a follow up to previous work in this area [30]. Experimental results will demonstrate the accuracy of the algorithm, which will be validated.

## 2. Image Processing Techniques and Filtration

The new developed algorithm is proposed with the purpose of detecting oocyte and polar bodies using images taken by a microscope camera. The detection is based on the microscope’s global reference to find the position and orientation of the polar body and oocyte center point. All the images used in this paper are of oocytes in the metaphase II state and taken from different resources. The oocyte was clearly denudated of all excess surrounding cumulus. The microscope was adjusted to have a clear view of the oocyte and surrounding polar body. The oocyte and polar body are commonly circular but may be presented as ovular/elliptical with eccentricity values similar to that of a circle [31]. The detection procedure has been divided into three stages; preprocessing, segmentation and feature extraction, which will be discussed in the following sections.

### 2.1. Preprocessing

The preprocessing procedure has three stages; conversion of the color, contrast enhancement and spatial filtering.

Digital color images represent the standard color model. The most common model for this is RGB which represents red, green and blue; these are the three principal components for each pixel of an image. The level of each of these components identifies the color for each image. A gray scale image is an image with an intensity ranging within black to white. Gray scale conversion from an RGB image would be achieved by Equation (1), which makes the level of each component equal by averaging the RGB for each pixel and computes the gray scale intensity.

After conversion of RGB image to grayscale image, the obtained images have low, medium or high gray values. These values will indicate how dark or light our image is. This information can be obtained from the image’s histogram.

(1) $$Greyscale_{Intensity\;}=\frac{R\left(x,y\right)+G\left(x,y\right)+B\left(x,y\right)}{3}$$

Gray scale images have different dynamic ranges based on the lowest and highest intensity level. Images with low contrast represented by low intensity of gray scale can be improved by raising the dynamic range to the highest potential determined by the bit depth of the images [32].

To raise the dynamic range of an 8-bit image from 0 to 255, the image intensity needs to be multiplied by an enhancement factor, which is represented in Equation (2):

(2) $$Ef=\frac{2^{n}-1\;}{Gray_{max}-Gray_{min}}$$

where *n* represents the number of bits for the gray scale image and 1 is deducted from the nominator to maintain the dynamic range within 0 to 255. Here gray scale images are 8-bit images. The enhanced image, *h*(*f*), is calculated using Equation (3) where the initial image’s minimum grey level is subtracted to zero the minimum intensity of the image and multiplied by the enhancement factor.

(3) $$h(f)\;=\left(g\left(f\right)-gray_{\min}\right)\left(Ef\right)$$

Spatial filtering is a filter application in a spatial domain. A filter mask is a neighborhood coefficient which is usually a 3 × 3 rectangle. The coefficients are varied and are selected based on the filter type.

The weighted average filter utilises the highest weight coefficient at the center of the filter mask and the rest of the coefficients are weighted inversely as a distance function [32]. The weighted average filter is a low-pass filter that suppresses low frequencies and eliminates noise, while minimizing blurring that results in edge preservation improvement [32]. The weighted average filter utilised is shown in Equation (4), which is obtained by dividing the mask into its summation. The process of applying the weighted average filter is obtained by Equation (5), which is the two-dimensional correlation filtering (*m* × *n*) of the initial image; this indicates the summation of the coefficient multiplied by the local pixel value to calculate the filtered value in the filter location center for each image pixel.

(4) $$w\left(x,y\;\right)=\frac{1}{16}\left[\begin{array}{ccc} 1 & 2 & 1\\ 2 & 4 & 2\\ 1 & 2 & 1 \end{array}\right]$$

(5a) R=w(1,1 )f(x+1,y+1)+w(1,0)f(x+1,y)+…+w(0,0)f(x,y)+…+w(−1,−1)f(x−1,y−1)

(5b) $$g\left(x,y\;\right)=\overset{\frac{m}{2}}{\underset{-\frac{m}{2}}{\sum }}\overset{\frac{n}{2}}{\underset{-\frac{n}{2}}{\sum }}w\left(u,v\right)f\left(x+u,y+v\right)=w\left(x,y\right)\times f\left(x,y\right)$$

**Figure 1:**
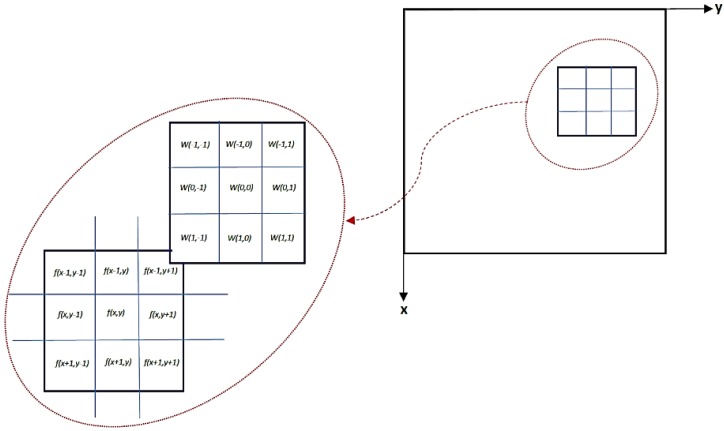
Mechanics of spatial filtering.

Figure 1 illustrates the mechanics of spatial filtering. The procedure contains simple movement of the filter mask from one point to another in an image. The response of each point is calculated by a predefined relationship. Weighted smoothing filters are more effective compared with the linear smoothing spatial filters.

### 2.2. Segmentation

The gradient thresholding of a single image involves the intensity vector derivatives regarding *x* and *y*. The edges within an image are the locations where there are sudden intensity changes [33]. Consequently, the gradient magnitude is employed to distinguish the edges by defining the threshold to find a location where the magnitude of the gradient is above the value of the threshold.

(6) $$\nabla f=\left[\begin{array}{c} G_{x}\;\\ G_{y} \end{array}\right]=\left[\begin{array}{c} \frac{\partial f}{\partial x}\\ \frac{\partial f}{\partial y} \end{array}\right]\;\;\|\nabla f\|=\sqrt{G_{x}{}^{2}+G_{y}{}^{2}}$$

(7) $$\theta =\tan^{-1}\left(\frac{G_{x}}{G_{y}}\right)\;\;\sin\theta =\frac{G_{y}}{\left\|\nabla f\right\|}\;\;\cos\theta =\frac{G_{x}}{\left\|\nabla f\right\|}$$

where (∇f) is the gradient magnitude which specifies the greatest changes in intensity at the pixel. The pixel position and orientation were computed from Equations (6) and (7).

### 2.3. Feature Extraction

**Figure 2:**
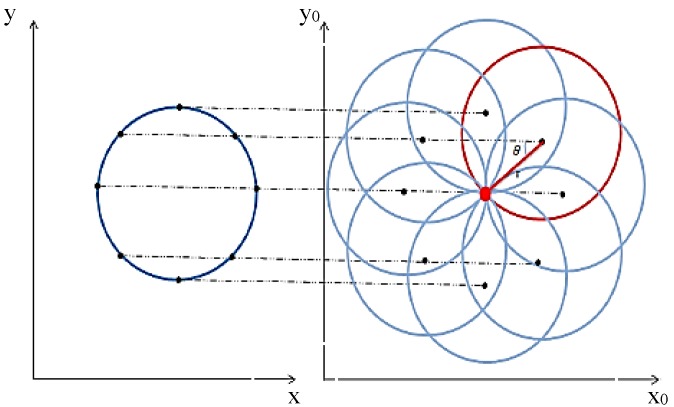
Circle illustration in an image and applying Hough transformation to a parameter space which is defined as an accumulator.

The final stage for image processing is feature extraction. In this section, the Hough transform method is employed, which is a reliable method for identifying parametric shapes within images. This method was initially proposed by Paul Hough for line detection [34]. In the current paper, a circular Hough transform method was used for feature extraction of the oocyte and polar body in the image as the shape of these is usually circular. A parametric circle equation is used and rearranged to calculate the center point voted by the edge points, that are planned to parametric space named as an accumulator, as shown in Figure 2.

As it is shown in Figure 2, the edge points direct a circular object to be recognized. Equation (7) shows the relationship between the orientation and gradient, which is employed to obtain the sin*θ* and cos*θ* for the vote received for each center point. The obtained vote for the position in the accumulator and the votes’ summation completes the mapping to the accumulator. As a result, in the presence of any circle in the image, circle detection and center point location would be obtained based on the center point votes which cluster in a small accumulator region and create a maximum region. The center point votes are obtained in the accumulator by employing a center point detection algorithm based on Equation (8).

(8) $$x_{0}=x-r\cos\theta \;\;y_{0}=y-r\sin\theta$$

The Weighted Hough Transform is a development of the Hough transform method which has a different convention for planning to the accumulator. This technique employs weighting coefficients for each vote. The gradient based system considers the magnitude of the gradient as the coefficient to plan the accumulator. Consequently, the votes for center point from the sharper edge would have higher weighting coefficients in comparison to the other votes received from blurred edges.

An elliptical fitting method is utilised to recognise an elliptical object to allow for cell morphological variance, not being a perfect circle. In this method, the center point has been calculated and radius ranges have been defined. The gradients of the image shown as *G_x_* and *G_y_* are calculated for the area, encapsulated in a square area. This is twice the maximum value for the radius surrounding the center point of an elliptical item existing in the image. The radius values which are indicated as a and b for an elliptical item, are determined based on the location of the maximum value in the accumulator. The values in addition to the value of the center point are employed to fit to the item using elliptical Equations (9):

(9) $$r=\sqrt{{\left(x-x_{0}\;\right)}^{2}+{\left(y-y_{0}\right)}^{2}}\;\;where\;\;x=x_{0}+a\cos\theta \;\;y=y_{0}+b\sin\theta$$

## 3. Detecting of Polar Body and Oocyte

**Figure 3:**
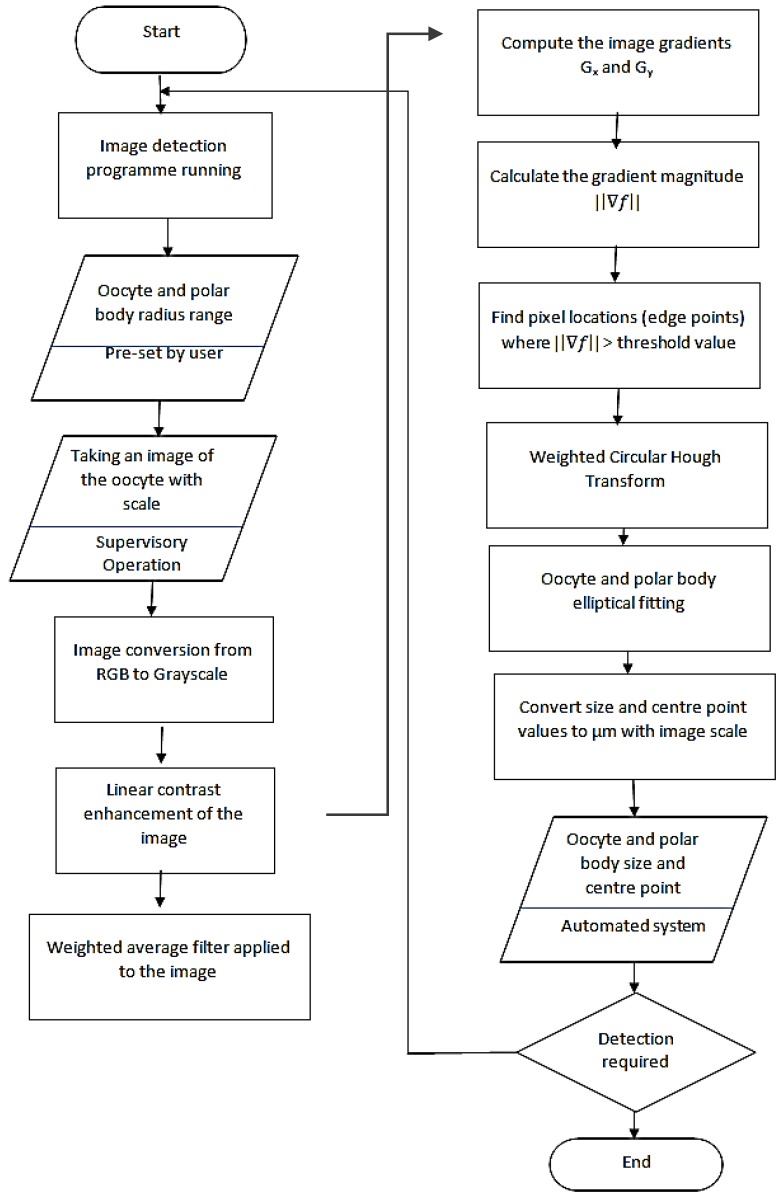
Detection algorithm process.

The design of the algorithm is based on the combination of the methods presented earlier in the previous section. The images used for the algorithm test were 8-bit JPEG-compressed RGB images received from different types of inverted microscope cameras. Then, all the images were converted to gray scale to save computational time for the subsequent steps. Linear contrast enhancement was employed for the purpose of raising the dynamic range of the images to achieve better and easier edge detection [35]. Figure 3 indicates the step by step detection procedure in the proposed algorithm.

A 5 × 5 weighted filter was applied to the images to minimize the presented noise in the image and also restrain the granularity presence in the polar body and oocyte. This filterization helps to reduce any possible false detection. The center point position of the polar body and oocyte were detected by employing the weighted circular Hough transform method. The obtained gradient magnitudes were used as the weighting coefficients. To minimize the calculation time, a gradient magnitude threshold is defined to limit the total number of the edge points based on the center point calculation.

As this algorithm is designed to be compatible for different types of oocyte, the radius range for both the oocyte and polar body is to be supplied by the user. This range was obtained from the literature for each iteration. The center point for both the oocyte and the polar body were extracted based on the developed algorithm. The microscope’s lens’ center point was taken as the main reference point of the image. The circular Hough transform (CHT) was utilized for the oocyte and polar body detection in any position and orientation in the image, which did not need to be in a close neighborhood area in the center of the image [36]. The elliptical Hough transform (EHT) is employed to detect an elliptical shape, which could be employed when the oocyte and/or polar body are this shape. It should be noted that the circular Weighted Hough Transform is also sufficient for detecting the center point, although EHT increases the accuracy of the detection in possible elliptical shapes.

The sizes of the polar body and oocyte were detected after obtaining the location of the center points. This center point locations were utilized within the elliptical fitting method, which was presented in the last section and recently developed for this specific application. The radius for the elliptical fitting for the polar body and oocyte detection was determined by considering the specified radius ranges by the user. This method does not need the edge of the polar body and oocyte to be identified and has a better fitting in comparison to a circular fitting for the elliptical and ovoid shapes; and consequently provides more accurate sizing.

**Figure 4:**
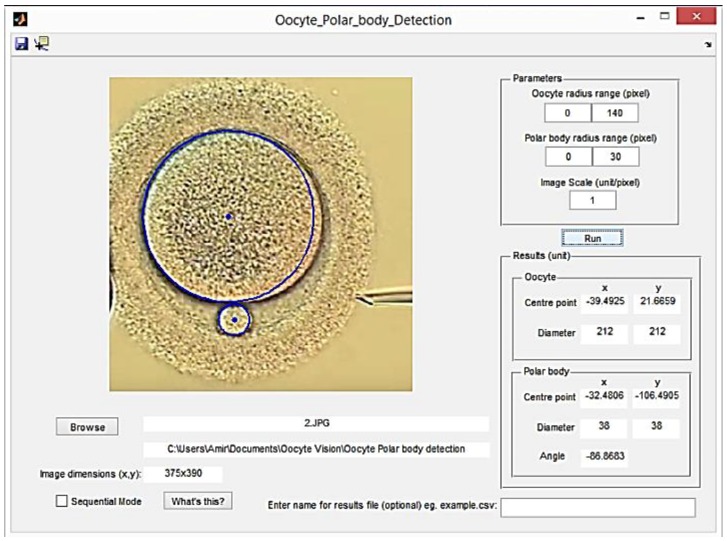
Graphical user interface (GUI) for the detection algorithm.

The algorithm is designed, via a graphical user interface (GUI), to get initial information from the operator and show the results based on the calculation. This GUI requires two parameter ranges which enable this algorithm to be compatible with different microscopes and different types of oocytes. Then, after pushing the ‘RUN’ button, the specification of the oocyte and polar body is as indicated in Figure 4.

**Figure 5:**
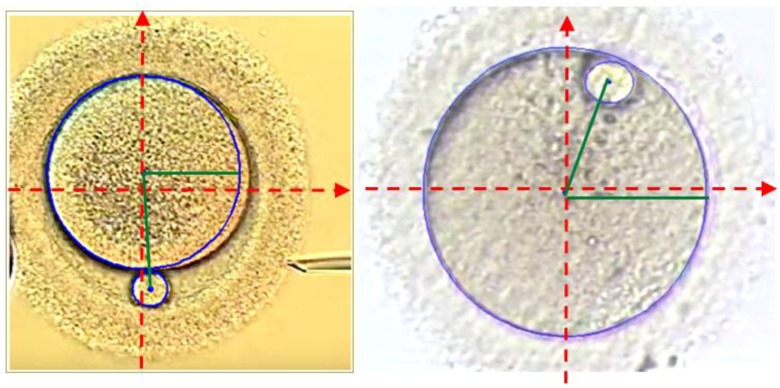
Processed images and detected oocyte and polar body as well as positional reports.

The output information received from the algorithm fed the proposed system about the initial location and positioning of the polar body and oocyte in global coordination. This information indicates the position and the diameter of the oocyte and polar body centers as well as the angle where polar body’s center positioned with respect to positive direction of the x-axis. The image center is the center of the microscope’s vision field. Figure 5 illustrates two random examples of the oocyte and polar body detections. The red lines indicates the image coordinate axis while the green line shows the angle between the polar body and oocyte centre points connection line with horizontal axis.

## 4. Experimental Results and Discussion

The experiments were conducted for various images sourced from numerous microscopes. The images were considered as input to the algorithm and the algorithm first detected the polar body and oocyte. If the polar body was not in the focal zone of the microscope, then the algorithm gave a message about not locating a polar body. After the confirmation of the correct recognition of the polar body and oocyte, the sizes of the oocyte and polar body were computed. The sizes of both the polar body and oocyte were measured manually using ImageJ algorithm (Version 1.52e, US National Institutes of Health, Bethesda, Maryland, MD, USA). ImageJ is a public domain image processing program developed for image analysis in biomedical applications.

The algorithm has been tested with different factors that may have an impact on the results. Eighty images have been tested for each major factor set. Table 1 compares 240 different images taken from different resources. The first row indicates the images taken from different microscopes with a different background color. It shows 100% correct detection on these types of sampling test. The second row images of different orientations of a polar body taken from the same microscope. The polar bodies are located in random locations in the perivitelline space. This test indicates the ability of the algorithm to detect orientation and position of the polar body in a space. A 100% successful detection has been reported for this set as well. The third row shows different microscope zooms. This test has been done to check the ability of the algorithm to detect the polar body and oocyte under different magnifications. This test also had a 100% success rate. The last row points out the failure of the algorithm in this type of image; this is because the images are blurred and the clarity of the image is poor.

The algorithm is capable of being adopted by different versions of the inverted microscopes. Existing extra cumulus cells are known as disturbance for the outcome of this algorithm and may cause some errors in the detection of the polar body due to its smaller size. However, the existence of these cells have not had any effect on oocyte detection.

The quality of an image is indicated by the behavior of the associated histogram. The good quality of an image is defined when the image is bright enough and has a good contrast. As a result, the histogram data is a measure of the brightness and contrast as well as an indication of the associated dynamic range.

Each of the analyzed categories of the images is indicated separately in the following sections. The results are demonstrated using two main methods. The first method illustrates the results of the detection and is shown as a naked image, its identity which is exhibited by the histogram, and the final detected result from the developed algorithm. The second method uses the box and whisker chart demonstration, which indicates the differences in the measurements made manually using ImageJ and the algorithm-based measurement.

### 4.1. Different Background Color

**Figure 6:**
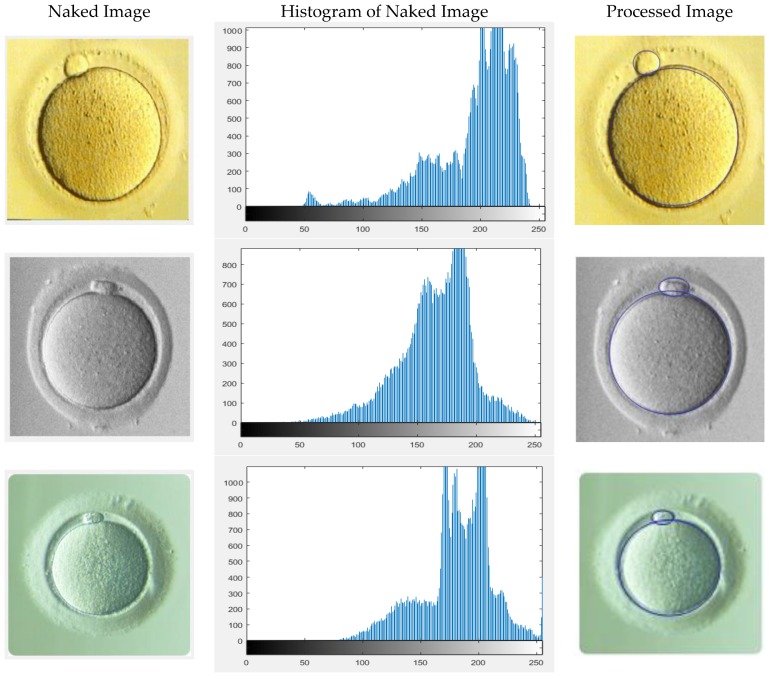
Different background color examined images.

Different microscopes operate with different vision background colors based on the type and purpose of the operation. This should be taken into consideration in designing a vision detection algorithm. The proposed algorithm is capable of detecting the polar body and the oocyte present in different background colors as demonstrated by the obtained results. Three different background colors are shown in Figure 6. The histogram of these images concentrate on high gray value but are not well-spread, which is due to poor contrast. As a result, during preprocessing of an image, contrast was increased. Consequently, this figure illustrates the correct detection of the oocyte and polar body in each image.

**Figure 7:**
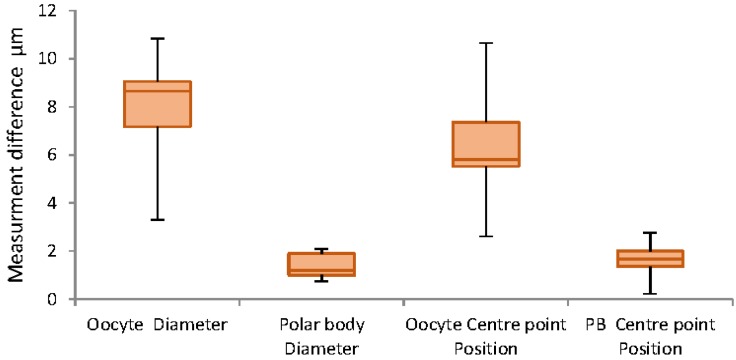
Whisker chart demonstrating differences in measurements in different background color.

Figure 7 illustrates the highest difference in manual and algorithm measurement belongs to oocyte diameter measurement. As the average oocyte diameter and polar body are 140 µm and 20 µm respectively, the corresponding maximum error for oocyte diameter measurements are approximately 5% ± 2% and 10%.

### 4.2. Different Polar Body Position and Orientation

**Figure 8:**
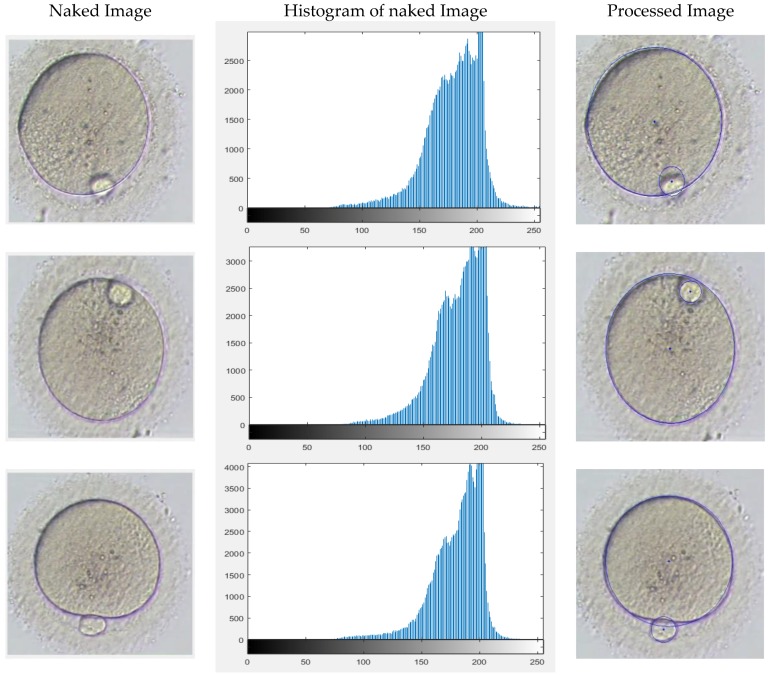
Different polar body positions examined images.

The oocyte is positioned on the petri dish at random, which results in the random positioning of its polar body. The proposed algorithm is capable of detecting the center point of the oocyte and that of its polar body, together with the line that connects these two points. The results confirm 100% successful detection of all of the 80 test images. Examples of the results are illustrated in Figure 8, where the detected images are circled by blue lines.

**Figure 9:**
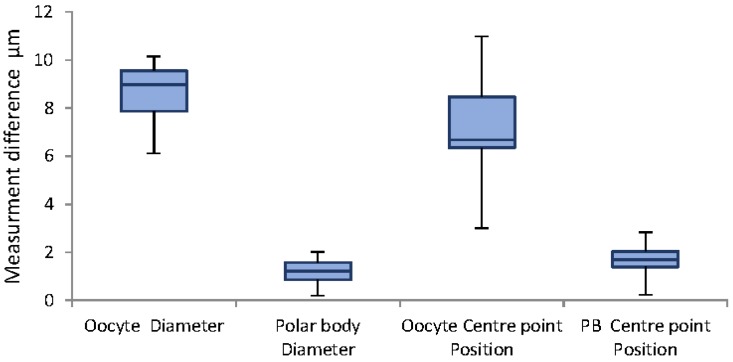
Whisker chart demonstrating differences in measurements in different polar body positions.

Validation with ImageJ algorithm confirms accuracy of the oocyte and polar body diameter and center point measurements, as shown in Figure 9. The figure illustrates that the majority of the differences for the oocyte diameter and positioning are less than 10 µm, and for polar body are approximately 2 µm. These are very satisfactory results for 100% correct detection containing less than 5% error in reporting the measurement.

### 4.3. Different Zoom and Intensity of the Images

**Figure 10:**
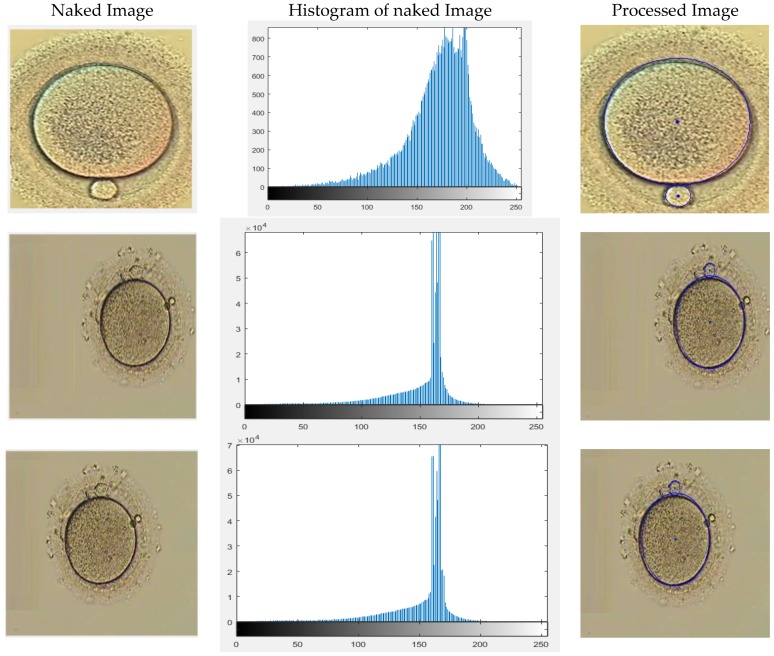
Different magnifications examined images.

Visualization of an oocyte can be improved by changing the magnification after random deposition of an oocyte under the focal zoom of an inverted microscope. Consequently, the proposed algorithm has the option of detecting the oocyte position under different Magnifications. Examples are given in Figure 10.

**Figure 11:**
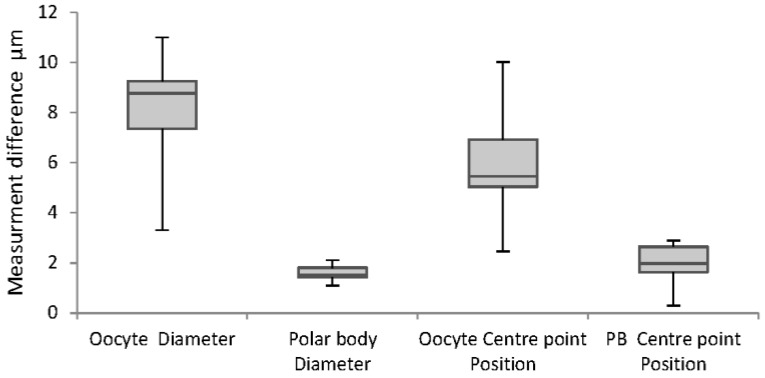
Whisker chart demonstrating differences in measurements in different magnifications.

The effect of different magnifications used by the microscope was another important factor that needed to be considered. In smaller magnifications the histograms show less contrast and less clarity of the oocyte, although the developed algorithm successfully fully detected the polar body and oocyte and the majority of the measurements are even less than 5% for oocyte and almost 10% for the polar body. The error for the polar body may be caused by smaller size in lower magnifications and also caused by human error in manual measurements. Figure 11 demonstrates the measurement obtained from the algorithm operation by changing the magnifications. The results indicate an average difference of 8.7 µm and 1.8 µm in oocyte and polar body measurements respectively.

### 4.4. Image Disturbance

**Figure 12:**
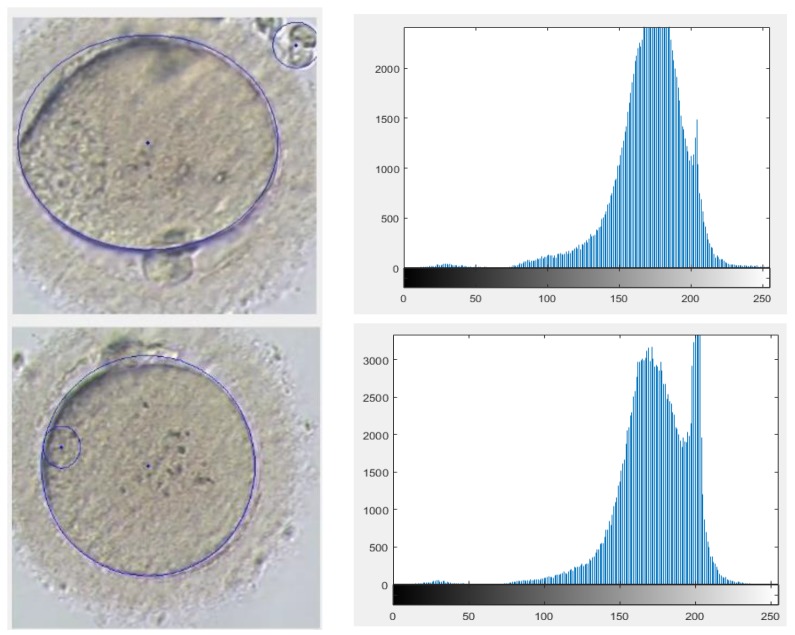

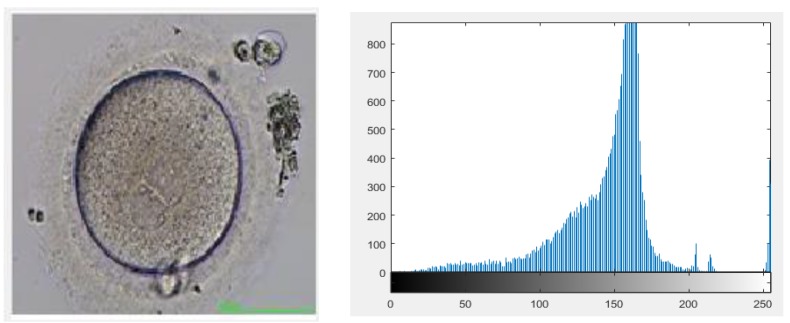
Different examined images including disturbance.

The oocyte should be positioned in the focal zone of the microscope without the presence of any cumulus. Oocyte denudation is the initial step after oocyte retrieval from the patient. If this denudation is not applied properly, the existing cumulus adversely affect the quality of the image and also that of the detection. This creates disturbance within the following image. In the proposed algorithm the detection success rate for the images that include cumulus is approximately 60%. Figure 12 demonstrates examples of such disturbance causing false detection.

**Figure 13:**
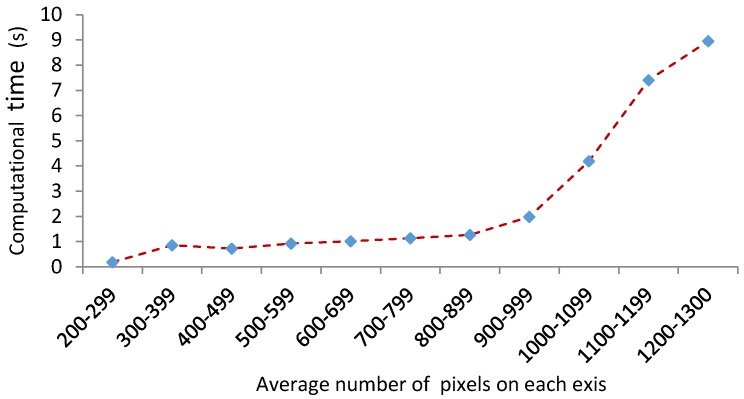
Computational time of the images based on the size of images.

Computational time was an important factor when developing this algorithm. The computational time is highly dependent on the quality and size of the image based on the pixels. After 80 experiments, the following graph was obtained showing the computational time. Figure 13 shows the graph of computational time with respect to the average size of the images. As it is indicated in the graph, the computational time for the images with the average amount of 900 pixels is 1 ± 0.2 s.

For the majority of the acceptable quality images, the computational time is 0.8 s, although if the number of pixels increases to more than 1000, the computational time suddenly significantly increases. This is a very acceptable computational time in comparison to similar image development algorithms which take 12 s.

**Figure 14:**
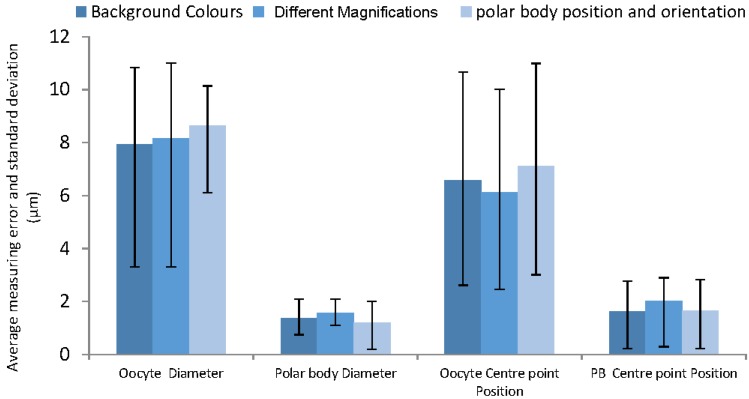
The measurement errors of the affecting factors.

The data obtained from the algorithm has been compared with the manual measurements using ImageJ. In ImageJ, the pixel size is calibrated with a reference in the image for measurements to be taken. The differences between the manual measurement and the algorithm measurement are indicated in Figure 14 for both oocyte and polar body diameter and position measurements. In this algorithm, the maximum reported error on measuring the oocyte and polar body diameters in the detection section is around 8 µm (~5% error) and 2 µm (~8% error) respectively. This indicates the considerable accuracy in dimension detection. On the other hand, this figure describes a high precision in center point detection which assists in positioning the samples after detection. Additionally, the reported errors in three considered factors are virtually similar, which indicates the independency of the algorithm from factors mentioned in Table 1.

## 5. Conclusions

In conclusion, this paper is mainly focused on the vision detection algorithm development for detecting the polar body and oocyte position and orientation, as well as measuring their sizes. This algorithm is designed to be integrated with a micromanipulation system designed for automated manipulation of metaphase II oocytes.

The developed algorithm is well-matched with various inverted microscopes with a range of imaging variables. This is the first report of utilizing the gradient-weighted Hough transform in the application of ICSI that also included elliptical fitting. This algorithm is able to detect the polar body and oocyte under different magnifications as well as with different background colors. The obtained results show a rate of 100% in detecting the oocyte and polar body. For the polar body, it drops to 60% in existing disturbances, which is caused by disruption in the area of the image and also morphological problems of the polar bodies. In terms of measurement, there was a 6% and a 9% error in the oocyte and polar body diameter measurements. In addition, there was less than 10-µm and a 2-µm error in the oocyte and polar body position measurements. The computational time is a considerable improvement, compared with similar algorithms, to 1 ± 0.2 s, which enables the robotic systems to operate in a shorter time domain and be closer to a real time application.

## Figures and Tables

**Table 1:** Comparison table for all tested images.

Description	Total Tested Images	Correct Oocyte Detection	Correct Polar Body Detection	Correct Oocyte Location Detection	Correct Polar Body Location Detection
Different background color	80	80	80	80	80
		100%	100%	100%	100%
Different position and orientation	80	80	80	80	80
		100%	100%	100%	100%
Different magnification of the images	80	80	80	80	80
		100%	100%	100%	100%
Existing disturbance	20	20	12	20	12
		100%	60%	100%	60%

## References

[1] Hogan B., Costantini F., Lacy E. (1986). Manipulating the Mouse Embryo: A Laboratory Manual.

[2] Hogg R.C., Bandelier F., Benoit A., Dosch R., Bertrand D. (2008). An automated system for intracellular and intranuclear injection. J. Neurosci. Methods.

[3] Wang Z., Latt W.T., Tan S.Y.M., Ang W.T. (2015). Visual servoed three-dimensional cell rotation system. IEEE Trans. Biomed. Eng..

[4] Lu Z., Zhang X., Leung C., Esfandiari N., Casper R.F., Sun Y. (2011). Robotic ICSI (intracytoplasmic sperm injection). IEEE Trans. Biomed. Eng..

[5] Feng L., Turan B., Ningga U., Arai F. Three dimensional rotation of bovine oocyte by using magnetically driven on-chip robot. Proceedings of the 2014 IEEE/RSJ International Conference on Intelligent Robots and Systems (IROS 2014).

[6] Zhao Q., Sun M., Cui M., Yu J., Qin Y., Zhao X. (2015). Robotic cell rotation based on the minimum rotation force. IEEE Trans. Autom. Sci. Eng..

[7] Nan Z., Xu Q. Multiple-cell recognition and path planning for robotic microinjection system. Proceedings of the 2017 36th Chinese Control Conference (CCC).

[8] Wang Z., Feng C., Ang W.T., Tan S.Y.M., Latt W.T. (2017). Autofocusing and polar body detection in automated cell manipulation. IEEE Trans. Biomed. Eng..

[9] Boukallel M., Gauthier M., Dauge M., Piat E., Abadie J. (2007). Smart microrobots for mechanical cell characterization and cell convoying. IEEE Trans. Biomed. Eng..

[10] Janocha H. Microactuators-principles, applications, trends. Proceedings of the VDE World Microtechnology Congress: Applications–Trends–Visions.

[11] Bellouard Y. (2009). Microrobotics: Methods and Applications.

[12] Leung C., Lu Z., Zhang X.P., Sun Y. (2012). Three-dimensional rotation of mouse embryos. IEEE Trans. Biomed. Eng..

[13] Nguyen B.V., Wang Q.G., Kuiper N.J., El Haj A.J., Thomas C.R., Zhang Z. (2010). Biomechanical properties of single chondrocytes and chondrons determined by micromanipulation and finite-element modelling. J. R. Soc. Interface.

[14] Mohanty S.K., Gupta P.K. (2004). Laser-assisted three-dimensional rotation of microscopic objects. R. Sci. Instrum..

[15] Shen Y., Fukuda T. (2014). State of the art: Micro-nanorobotic manipulation in single cell analysis. Robot. Biomim..

[16] Chronis N., Lee L.P. Polymer mems-based microgripper for single cell manipulation. Proceedings of the 17th IEEE International Conference on Micro Electro Mechanical Systems (MEMS).

[17] Jager E.W., Inganäs O., Lundström I. (2000). Microrobots for micrometer-size objects in aqueous media: Potential tools for single-cell manipulation. Science.

[18] Ichikawa A., Sakuma S., Shoda T., Arai F., Akagi S. On-chip enucleation of oocyte using untetherd micro-robot with gripping mechanism. Proceedings of the 2013 International Symposium on Micro-NanoMechatronics and Human Science (MHS).

[19] Zhang Y., Tan K.K., Huang S. (2009). Vision-servo system for automated cell injection. IEEE Trans. Ind. Electron..

[20] Shin Y.K., Kim Y., Kim J. Automated microfluidic system for orientation control of mouse embryos. Proceedings of the 2013 IEEE/RSJ International Conference on Intelligent Robots and Systems (IROS).

[21] Otsu N. (1979). A threshold selection method from gray-level histograms. IEEE Trans. Syst. Man Cybern..

[22] Wang Y., Zhao X., Zhao Q., Lu G. Illumination intensity evaluation of microscopic image based on texture information and application on locating polar body in oocytes. Proceedings of the China Automation Conference.

[23] Chen D., Sun M., Zhao X. (2016). Oocytes polar body detection for automatic enucleation. Micromachines.

[24] Zhao Q., Cui M., Zhang C., Yu J., Sun M., Zhao X. Robotic enuleation for oocytes. Proceedings of the 9th IEEE International Conference on Nano/Micro Engineered and Molecular Systems (NEMS).

[25] Sun Y., Nelson B.J. (2002). Biological cell injection using an autonomous microrobotic system. Int. J. Robot. Res..

[26] Du Q., Zhang Q., Tian L., Wu Z. Object detection and tracking for a vision guided automated suspended cell injection process. Proceedings of the 2010 International Conference on Mechatronics and Automation (ICMA).

[27] Blake A., Isard M. (2012). Active Contours: The Application of Techniques from Graphics, Vision, Control Theory and Statistics to Visual Tracking of Shapes in Motion.

[28] Diaz J.F. (2011). Ros-Drill Automation: Visual Feedback Control and Rotational Motion Tracking. Master’s Thesis.

[29] Hajiyavand A.M., Saadat M., Bedi A.-P.S. Polar body detection for ICSI cell manipulation. Proceedings of the International Conference on Manipulation, Automation and Robotics at Small Scales (MARSS).

[30] Rienzi L., Balaban B., Ebner T., Mandelbaum J. (2012). The oocyte. Hum. Reprod..

[31] Al-amri S.S., Kalyankar N., Khamitkar S. (2010). Linear and non-linear contrast enhancement image. Int. J. Comput. Sci. Netw. Secur..

[32] Rafael Gonzalez C., Woods R. (2002). Digital Image Processing.

[33] Priya S., Kumar T.A., Paul V. A novel approach to fabric defect detection using digital image processing. Proceedings of the 2011 International Conference on Signal Processing, Communication, Computing and Networking Technologies (ICSCCN).

[34] Hough P.V. (1962). Method and Means for Recognizing Complex Patterns. U.S. Patent.

[35] Caponetti L., Castellano G., Basile M.T., Corsini V. (2014). Fuzzy mathematical morphology for biological image segmentation. Appl. Intell..

[36] Hamarneh G., Althoff K., Abu-Gharbieh R. (1999). Automatic Line Detection.

